# Suppression of Medulloblastoma Lesions by Forced Migration of Preneoplastic Precursor Cells with Intracerebellar Administration of the Chemokine Cxcl3

**DOI:** 10.3389/fphar.2016.00484

**Published:** 2016-12-16

**Authors:** Manuela Ceccarelli, Laura Micheli, Felice Tirone

**Affiliations:** Genetic Control of Development, Institute of Cell Biology and Neurobiology – National Research Council, Fondazione Santa LuciaRome, Italy

**Keywords:** Cxcl3, chemokines, migration, preneoplastic lesions, medulloblastoma, differentiation and reprogramming, cerebellum development, cancer therapy

## Abstract

Medulloblastoma (MB), tumor of the cerebellum, remains a leading cause of cancer-related mortality in childhood. We previously showed, in a mouse model of spontaneous MB (*Ptch1*^+/-^/*Tis21*^-/-^), that a defect of the migration of cerebellar granule neuron precursor cells (GCPs) correlates with an increased frequency of MB. This occurs because GCPs, rather than migrating internally and differentiating, remain longer in the proliferative area at the cerebellar surface, becoming targets of transforming insults. Furthermore, we identified the chemokine Cxcl3 as responsible for the inward migration of GCPs. As it is known that preneoplastic GCPs (pGCPs) can still migrate and differentiate like normal GCPs, thus exiting the neoplastic program, in this study we tested the hypothesis that pGCPs within a MB lesion could be induced by Cxcl3 to migrate and differentiate. We observed that the administration of Cxcl3 for 28 days within the cerebellum of 1-month-old *Ptch1*^+/-^/*Tis21*^-/-^ mice, i.e., when MB lesions are already formed, leads to complete disappearance of the lesions. However, a shorter treatment with Cxcl3 (2 weeks) was ineffective, suggesting that the suppression of MB lesions is dependent on the duration of Cxcl3 application. We verified that the treatment with Cxcl3 causes a massive migration of pGCPs from the lesion to the internal granular layer, where they differentiate. Thus, the induction of migration of pGCPs in MB lesions may open new ways to treat MB that exploit the plasticity of the pGCPs, forcing their differentiation. It remains to be tested whether this plasticity continues at advanced stages of MB. If so, these findings would set a potential use of the chemokine Cxcl3 as therapeutic agent against MB development in human preclinical studies.

## Introduction

Medulloblastoma (MB), a neuroepithelial tumor developing in the cerebellum, is the most common pediatric brain cancer and represents about 20% of all cerebral childhood tumors (Roberts et al., 1991; Louis et al., 2007). The current therapy of MB involves preliminary surgery, radiation, and chemotherapy. This standard multimodality treatment allows acceptable survival rates, but patients suffer devastating morbidity, such as permanent neurocognitive dysfunctions (Gottardo and Gajjar, 2008) and secondary malignancies (Goldstein et al., 1997). Thus, new, less toxic and more targeted, therapy options are needed.

About 30% of MBs originate from the granule neuron precursor cells (GCPs) located in the external granular layer (EGL) at the surface of the developing cerebellum, in consequence of the proliferative hyperactivation of the Sonic Hedgehog (Shh) pathway (Schüller et al., 2008; Yang et al., 2008; Gibson et al., 2010; Northcott et al., 2012). In physiological conditions, the GCPs intensely proliferate postnatally in the EGL mitogenic niche, under the stimulus of Shh, and exit the cell cycle and differentiate as a result of migrating inward to molecular and internal granular layers (ML and IGL, respectively) (Choi et al., 2005). Given that the prolonged mitotic activity makes the GCPs especially susceptible to cell transformation (Wang and Zoghbi, 2001), the rate of migration, by regulating the duration of the period during which GCPs remain proliferating in the EGL, can impact the incidence of MBs, as we and others have shown (Farioli-Vecchioli et al., 2012, 2013; Haag et al., 2012). In fact, our recent study demonstrated that a new MB Shh-type mouse model, which lacks the MB-suppressor gene *Tis21* (*Ptch1*^+/-^/*Tis21*^-/-^), develops MBs with high frequency in consequence of a defect of migration of the GCPs out of the EGL (Farioli-Vecchioli et al., 2007, 2012). This defect of migration is caused by downregulation of the chemokine Cxcl3, whose promoter is directly activated by Tis21 (Farioli-Vecchioli et al., 2012). We also revealed the ability of the chemokine Cxcl3 to cell autonomously induce the migration of the GCPs out of the EGL and, remarkably, to reduce the area of MB lesions in cerebellar slices of the MB *Ptch1*^+/-^/*Tis21*^-/-^ mouse model (Farioli-Vecchioli et al., 2012).

It is known that the preneoplastic GCPs (pGCPs) within MB lesions, although they are able to generate a tumor when transplanted, can still migrate and differentiate like normal GCPs (Yang et al., 2008; Kessler et al., 2009). Thus, we reasoned that if pGCPs are induced to migrate out of the lesion at the surface of the cerebellum, they may differentiate and exit the neoplastic program.

Therefore, the chief aim of this study was to assess the feasibility of using *in vivo* the pro-migratory chemokine Cxcl3 as an agent able to reduce the frequency of tumor lesions and, hence, to contrast the development of MB.

## Materials and Methods

### Mice

The *Tis21* knockout mice were previously generated in the C57BL/6 strain as described (Park et al., 2004), by inserting the neomycin resistance cassette within exon II of the *Tis21* gene. *Patched1* heterozygous mice (*Ptch1*^+/-^) were produced in CD1 background through deletion of exons 6 and 7 (Hahn et al., 1998). The crossing of *Ptch1*^+/-^ with *Tis21*^-/-^ mice generated *Ptch1*^+/-^/*Tis21*^-/-^ double-mutant mice, which were interbred for at least six generations to obtain an isogenic progeny.

Genotyping of *Ptch1*^+/-^/*Tis21*^-/-^ mice was routinely performed by PCR analysis, using genomic DNA from tail tips as described (Farioli-Vecchioli et al., 2012).

Experiments were performed with either male or female mice and all animal procedures were accomplished according to the current European Ethical Committee guidelines (directive 2010/63/EU; authorization DM 307/2013-B of the Italian Ministry of Health).

### Brain Infusion

All mice involved in our study (*n* = 30) were anesthetized (80/80/10 mg/kg tiletamine HCl/zolazepam HCl/xylazine, i.p.) and implanted with an Alzet osmotic minipump at postnatal day 30 (P30), as described (Liao et al., 2008). Briefly, an Alzet 30-g infusion cannula (length 1.5 mm; brain infusion kit 3; Durect Corp., Cupertino, CA, USA) was implanted into the subarachnoid space above the rostral, dorsal cerebellum, 4 mm caudal to lambda at midline. The Alzet minipump (1004, which delivers 0.11 μl/h for 4 weeks; Durect Corp.) was filled with recombinant Cxcl3 (100 μl of a solution at 20 μg/ml; 5568-CA-025/CF, R&D Systems, Minneapolis, MN, USA) or with the vehicle (CSF, cerebrospinal fluid solution, as per Durect Corp. protocol: 148 mM NaCl, 3 mM KCl, 1.4 mM CaCl_2_, 0.8 mM MgCl_2_, 8 mM Na_2_HPO_4_, 0.2 mM NaH_2_PO_4_) and placed between the scapulas. The cannula was secured with cyanoacrylate and the skin was closed with sutures. An intraperitoneal injection of 0.9% normal saline was given after surgery to prevent post-operative dehydration. Animals were monitored post-operatively until mobile, and they were observed daily until the end of treatment (2 or 4 weeks). The number of mice for each experimental group is summarized in **Table 1**.

**Table 1:** Frequency of medulloblastoma lesions after Cxcl3 treatment.

Treatment	Mice with MB lesions (%, #)		Number of MB lesions per cerebellum^∗^ (Mean ± SEM)	
28 days in 1-month-old *Ptch1*^+/-^/*Tis21*^-/-^ mice	CSF	Cxcl3	CSF	Cxcl3
	62.5 (5/8)	0.00 (0/8)	0.62 ± 0.1	0.0 ± 0.0
n (mice) analyzed	8	8	8	8
Probability (*p*)	0.007 (Chi square test)		0.0042 (Student’s *t-*test)	

14 days in 1-month-old *Ptch1*^+/-^/*Tis21*^-/-^ mice	CSF	Cxcl3	CSF	Cxcl3
	42.8 (3/7)	28.5 (2/7)	0.42 ± 0.2	0.28 ± 0.1
n (mice) analyzed	7	7	7	7
Probability (*p*)	0.57 (Chi square test)		0.61 (Student’s *t-*test)	

*^∗^Average number in lesioned and non-lesioned mice.*

### Bromodeoxyuridine Treatment of Mice

The pGCPs entering S-phase were detected 1 h after an injection of bromodeoxyuridine (BrdU) (95 mg/kg, i.p.), according to existing protocols (Canzoniere et al., 2004; Qiu et al., 2010).

The pGCPs migrating from the lesions to the inner layers were visualized 5 days after a single injection of BrdU (95 mg/kg, i.p.) in P39 mice.

### Immunohistochemistry

At the end of treatment, the mice were euthanized under anesthesia by transcardiac perfusion with 4% paraformaldehyde in PBS and the cerebella were dissected and cryoprotected in 30% sucrose in PBS. Samples were embedded in Tissue-Tek OCT (Sakura Finetek, Torrance, CA, USA) and free-floating sagittal sections of 40 μm were cut serially on a rotary microtome. To detect the BrdU incorporation, the sections were treated with 2N HCl 45 min at 37°C and then with 0.1 M sodium borate buffer, pH 8.5, for 10 min. The samples were reacted with a rat monoclonal antibody against BrdU (AbD Serotec, Raleigh, NC, USA; MCA2060; 1:300) and a mouse monoclonal antibody raised against NeuN (Merck Millipore, Billerica, MA, USA; MAB377; 1:100), followed by donkey anti-rat tetramethylrhodamine isothiocyanate (TRITC)-conjugated and donkey anti-mouse Cy2-conjugated secondary antibodies (Jackson ImmunoResearch, West Grove, PA, USA; 1:200). The samples were then counter-stained by Hoechst 33258 (Sigma-Aldrich, St. Louis, MO, USA; 1 mg/ml in PBS) to visualize the ML and IGL.

Photomicrographs of the immunostained sections were generated by Laser Scanning Confocal Microscopy with a TCS SP5 microscope (Leica Microsystems) and analyzed by the I.A.S. software (Delta Sistemi, Rome, Italy).

### Lesion Quantification and Cell Migration Assay

All cerebella were stained for BrdU incorporation using fluorescent methods, as described above, to identify the presence of lesions. For each cerebellum were examined 12 sections spaced with intervals of 400 μm, in order to allow a representative sampling.

The pGCPs migrating from the lesions were identified as BrdU-labeled cells in MB lesion and neighboring layers (i.e., ML and IGL) and were counted as percentage ratio to the total number of BrdU^+^ cells in the lesion and layers, in 10 adjacent sagittal sections per lesion. We examined all lesions present in each 6-week-old mouse analyzed, whose number for each treatment is indicated in **Table 1**.

### Statistical Analysis

The Chi square test was used to compare the percentage of positivity for MB lesions in CSF-treated mice versus Cxcl3-treated mice. Data of migration and differentiation of pGCPs were calculated as the mean ± the standard error of the mean (SEM) and compared by the Student’s *t*-test. All statistical tests were two-sided and a value of *p* < 0.05 was considered statistically significant.

## Results

**FIGURE 1:**
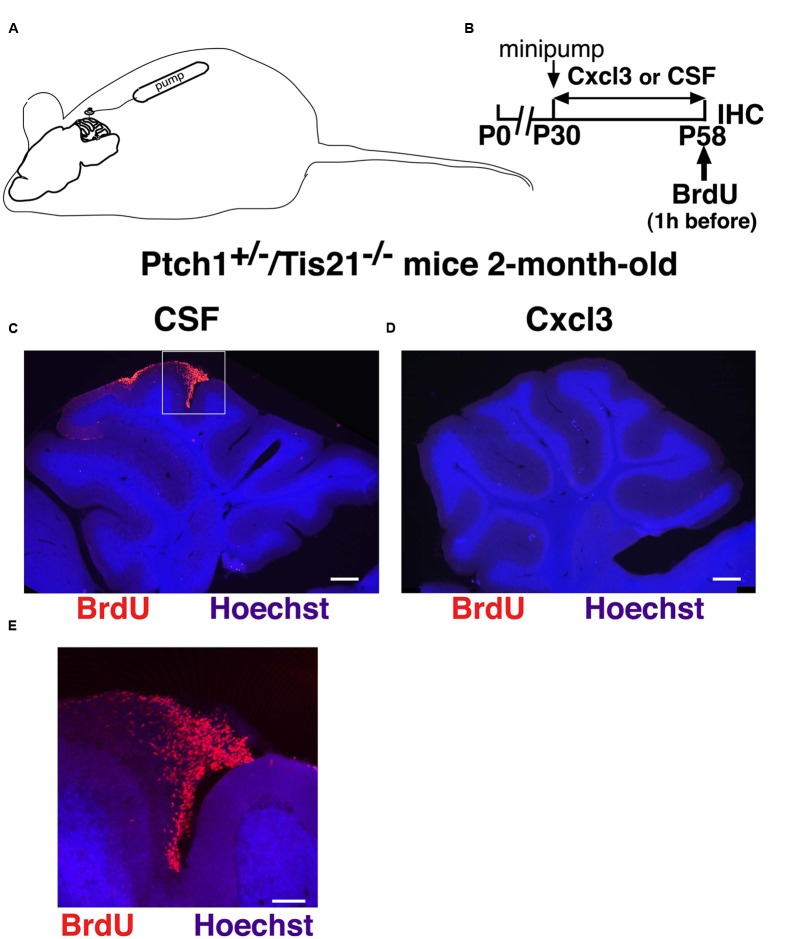
**Administration of Cxcl3 for 28 days suppresses the development of MB lesions. (A)** Scheme of minipump implant and **(B)** treatment timeline: 1-month-old *Ptch1*^+/-^/*Tis21*^-/-^ mice were treated for 4 weeks with Alzet minipumps filled with recombinant Cxcl3 or with CSF alone; a single BrdU injection was performed 1 h before analysis. **(C,D)** Representative images of cerebellar sagittal sections from 2-month-old *Ptch1*^+/-^/*Tis21*^-/-^ mice treated with CSF **(C)** or Cxcl3 **(D)**, with or without an MB lesion, respectively. Nuclei were stained with Hoechst 33258 and lesions were identified by the presence of BrdU^+^ pGCPs (red). Scale bar, 300 μm. **(E)** Higher magnification of the preneoplastic lesion indicated in **(C)** (white box). Scale bar, 100 μm.

The chemokine Cxcl3 or the vehicle (CSF) was administered by Alzet osmotic minipumps in the cerebella of the *Ptch1*^+/-^/*Tis21*^-/-^ mice, a mouse model that we have previously generated, which develops Shh-dependent MBs at high frequency (80%) (Farioli-Vecchioli et al., 2012). The treatment lasted 4 weeks and started in 1-month-old *Ptch1*^+/-^/*Tis21*^-/-^ mice, i.e., at a stage when MB lesions, generated by pGCPs, have already started to form and represent MB at its initial expansion (Kim et al., 2003; Farioli-Vecchioli et al., 2012). The lesions were identified by visualizing the proliferating pGCPs as BrdU^+^ cells, 1 h after an injection of BrdU (**Figures 1A,B**). As shown in **Figures 1C–E** and in **Table 1**, we observed that in 2-month-old *Ptch1*^+/-^/*Tis21*^-/-^ mice, previously treated with Cxcl3 for 28 days, the percentage of mice presenting cerebellar lesions was 0.0%, whereas it was 62.5% in CSF-treated mice (*p* = 0.007 Chi square test; *n* = 8 mice treated with Cxcl3 and *n* = 8 mice treated with CSF). Correspondingly, the average number of MB lesions per cerebellum resulted 0.0 ± 0.0 in Cxcl3-treated and 0.62 ± 0.1 in vehicle-treated mice (*p* = 0.0042 Student’s *t*-test). Thus, the chronic infusion of the chemokine Cxcl3 into the cerebellum of the high frequency MB mouse model *Ptch1*^+/-^/*Tis21*^-/-^ prevents and/or suppresses the development of MB lesions.

**FIGURE 2:**
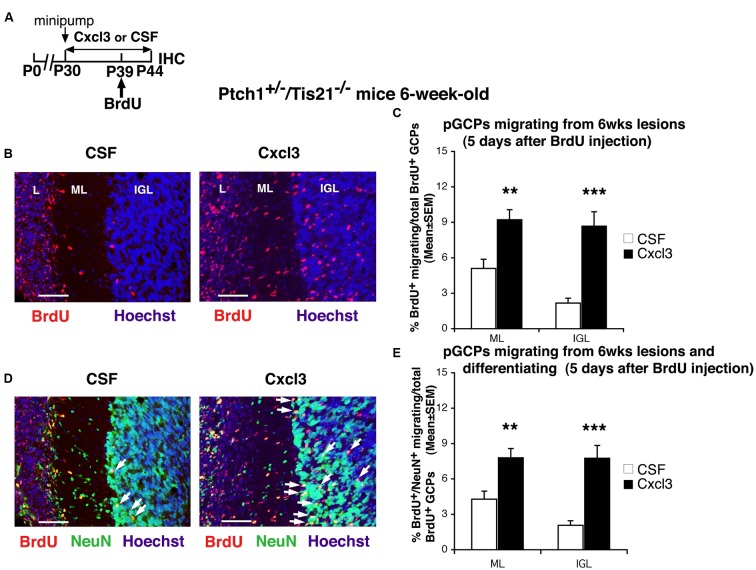
**Cxcl3 delivered *in vivo* in the cerebellum has a strong pro-migratory effect on pGCPs. (A)** Scheme of treatment: 1-month-old *Ptch1*^+/-^/*Tis21*^-/-^ mice were treated for 14 days with Alzet minipumps filled with recombinant Cxcl3 or with CSF alone; mice received a single BrdU injection at P39 and were analyzed 5 days after (P44). **(B)** Representative confocal images of pGCPs migrating outside the lesions, identified at 6 weeks of age as BrdU^+^ cells (red) in the *Ptch1*^+/-^/*Tis21*^-/-^ mice treated with CSF or Cxcl3. Sections are counterstained with Hoechst 33258 to visualize the ML and the IGL. Scale bar, 50 μm. L: lesion. **(C)** The pGCPs migrating from lesions were quantified as mean ± SEM percentage ratio of BrdU^+^ cells present within the ML or the IGL area neighboring each lesion to the total number of BrdU^+^ cells in lesion, ML and IGL. All lesions present in each mouse cerebellum were analyzed. CSF *n* = 3 lesions (seven mice), Cxcl3 *n* = 2 lesions (seven mice). ^∗∗^*p* < 0.01, ^∗∗∗^*p* < 0.001, Student’s *t*-test. **(D)** The same confocal sections of **(B)** are shown with pGCPs double labeled for BrdU^+^NeuN^+^ (red and green, respectively) and counterstained with Hoechst 33258 to visualize the ML and the IGL. Scale bar, 50 μm. White arrows indicate some of the BrdU^+^NeuN^+^ cells. **(E)** Quantification of the differentiated pGCPs [shown in graph **(C)** as BrdU^+^], analyzed as mean ± SEM percentage ratio of BrdU^+^NeuN^+^ cells present within the ML or the IGL area neighboring each lesion to the total number of BrdU^+^ cells in lesion, ML and IGL. CSF *n* = 3 lesions (seven mice), Cxcl3 *n* = 2 lesions (seven mice). ^∗∗^*p* < 0.01, ^∗∗∗^*p* < 0.001, Student’s *t*-test.

As a next step, we sought to define whether this striking anti-lesion effect was dependent on the duration of Cxcl3 application. We analyzed the frequency of MB lesions by treating 1-month-old *Ptch1*^+/-^/*Tis21*^-/-^ mice for a shorter period (14 days) with the same Alzet minipumps filled with recombinant Cxcl3 or with CSF (**Figure 2A**). At this time no significant differences were detected between Cxcl3-treated and CSF-treated mice in the percentage of MB lesions or in the number of lesions per cerebellum (see **Table 1**), indicating that 2 weeks of Cxcl3 treatment are not sufficient to counteract the development of the MB lesions.

Thus, we reasoned that the absence of lesions in mice treated for a month with Cxcl3 could depend on the protracted period of forced migration out of the lesion of the pGCPs induced by the chemokine. Then, to verify the effect of Cxcl3 on pGCPs migration and differentiation, we analyzed the percentage of pGCPs migrated outside lesions in 6-week-old *Ptch1*^+/-^/*Tis21*^-/-^ mice previously treated with Cxcl3 or CSF for 14 days, labeled with one injection of BrdU 5 days before analysis, and following their migration to the ML and IGL (**Figures 2A–C**). pGCPs were double labeled (BrdU^+^NeuN^+^) to monitor also their differentiation (**Figures 2D,E**); in fact NeuN marks post-mitotic, terminally differentiated, cerebellar granule neurons (Weyer and Schilling, 2003). In Cxcl3-treated mice we observed an evident increase in the percentage of BrdU^+^ pGCPs migrated from the lesion to the ML and IGL (about 80% and fourfold, respectively), relative to control mice (percentage of BrdU^+^/total BrdU^+^ in Cxcl3-treated vs. CSF-treated mice, *p* = 0.0013 in ML and *p* = 0.00008 in IGL; **Figures 2B,C**). Almost all of the BrdU^+^ pGCPs migrated to the ML and IGL resulted terminally differentiated (85 and 90%, respectively; percentage of BrdU^+^NeuN^+^/total BrdU^+^ cells in Cxcl3-treated vs. CSF-treated mice, *p* = 0.002 in ML and *p* = 0.00008 in IGL; **Figures 2D,E**).

We conclude that Cxcl3 delivered *in vivo* in the cerebellum exerts a powerful pro-migratory effect on pGCPs – continuously forcing them to leave lesions – accompanied by a pro-differentiative effect resulting from the exit of pGCPs out of the mitogenic area at the cerebellar surface.

## Discussion

Current cancer therapies target proliferation and survival of tumor cells, mainly using toxic chemicals. Instead, in this study we tested the feasibility to reduce the frequency of MB lesions by forcing the preneoplastic granule neuron precursor cells (pGCPs) to migrate out of the lesions at the surface of the cerebellum and differentiate, withdrawing from the tumor program, by means of an intracerebellar treatment with the chemokine Cxcl3.

Some chemokines have been shown to be expressed in cerebellum, such as Cxcl2 (Rossi and Zlotnik, 2000), or Cxcl12 and Cxcl3, which inhibit (Ma et al., 1998) or promote (Farioli-Vecchioli et al., 2012), respectively, the migration of the GCPs out of the EGL.

Moreover, we have recently shown by real time-PCR and by *in situ* analysis that the chemokine Cxcl3 is expressed in normal as well as in preneoplastic GCPs in MB lesions and also in granule neurons in the IGL; furthermore, we have ascertained the expression of the Cxcl3 receptor (CXCR2) in GCPs (Farioli-Vecchioli et al., 2012).

To increase the levels of Cxcl3 *in vivo* in the pGCPs of the high frequency MB mouse model *Ptch1*^+/-^/*Tis21*^-/-^, we treated chronically 1-month-old mice with Cxcl3 in cerebellum by implantation of Alzet osmotic minipumps. As a result, after 1 month of intracerebellar administration of the chemokine, the tumor lesions disappeared or were prevented completely. Since the tumor lesions present at 2 months of age in *Ptch1*^+/-^/*Tis21*^-/-^ mice develop invariably into a MB (Farioli-Vecchioli et al., 2012), these data represent a proof of concept that the treatment with Cxcl3 can reduce MB formation. This suggests that the timing chosen to start the treatment, i.e., when the lesions have already started to develop (1 month after birth), is appropriate. Nevertheless, since a shorter treatment does not attain a significant reduction of lesion number, this study also suggests that the reduction of the frequency of MB lesions is dependent on the duration of Cxcl3 application. The observed delay in Cxcl3 effect on lesion frequency after 14 days of treatment may depend on the time required for diffusion and influence of Cxcl3 on brain microenvironment. In fact, in isolated cerebellar slices from MB *Ptch1*^+/-^/*Tis21*^-/-^ mice we observed a rapid decrease of the area of MB lesions within 5 days (Farioli-Vecchioli et al., 2012).

Notably, we showed in this report that the absence of lesions in the mice treated with Cxcl3 was correlated with increased migration out of the lesion and with differentiation of the pGCPs following the exposure to the chemokine. In fact, in mice treated with Cxcl3 for 2 weeks we observed a significant increase in the percentage of differentiated pGCPs (BrdU^+^NeuN^+^) migrated out of the lesion to the ML and IGL, relative to control mice. Interestingly, the percentage of BrdU^+^ pGCPs migrated out the lesion to either the ML or IGL of CSF-treated *Ptch1*^+/-^/*Tis21*^-/-^ mice matched the values of the pGCPs migration previously observed without minipumps (Farioli-Vecchioli et al., 2012), thus ruling out a possible effect of the CSF treatment on the ability of pGCPs to migrate out of lesions.

As a whole, our data confirm the importance of the timing of migration of GCPs in the MB pathogenesis and demonstrate, for the first time, the possibility to prevent or fully inhibit MB development by the migration-promoting action of Cxcl3. This chemokine, mimicking the morphogenetic migration process of GCPs from EGL to IGL, forces the pGCPs to differentiate and exit the neoplastic program, instead of controlling their proliferation. Moreover, it is worth mentioning that Cxcl3 is devoid of any intrinsic action on the differentiation or proliferation of the GCPs (Farioli-Vecchioli et al., 2012). Interestingly, a recent report showed that Cxcl3 promotes the proliferation and tumorigenesis of hepatocarcinoma cancer stem cells (Zhang et al., 2016). This, however, it not surprising, as it is quite common to have cell-specific effects among chemokines, including Cxcl3, and also considering that GCPs, although of epithelial origin as epatic cells, are not stem cells. As an example of different cell specificities, Cxcl3 has different actions on endothelial-derived blood cells, since it promotes the arrest of monocytes (Smith et al., 2005), while acts as chemoattractant for neutrophils to areas of brain injury (Szmydynger-Chodobska et al., 2009). Thus, different pathways are likely to be involved. In this regard, a recent analysis of genomic pathways in the *Ptch1*^+/-^/*Tis21*^-/-^ mouse model suggests that the chemotaxis mechanism of the GCPs by the Cxcl3-Cxcr2 axis may be clathrin-dependent (Gentile et al., 2016).

Further studies will be necessary to assess whether Cxcl3 will efficiently reduce the frequency of MBs also starting at later stages of MB development (after 1 month of age). And it will also be necessary to test whether the induction of migration at later stages could be less beneficial because of an increasing refractoriness of the pGCPs to leave the neoplastic program and differentiate, thus increasing the possibility of metastases.

If Cxcl3 will be validated as a therapeutic agent also at later stages of tumorigenesis, this will improve the possibility to use the chemokine in human therapy. Moreover, given that the control of migration of neural precursors is operative also in other neural tumors, this potential therapy may have wider application.

## Author Contributions

MC and FT designed the experiments and interpreted the data; MC, LM, and FT carried out the experimental work; MC and FT wrote the paper; MC, LM, and FT are responsible for accuracy and integrity of any part of the work.

## Conflict of Interest Statement

A patent was filed by the National Research Council on the possible use of the chemokine Cxcl3 in medulloblastoma therapy. The authors declare that the research was conducted in the absence of any commercial or financial relationships that could be construed as a potential conflict of interest.
